# Relationship between serum vitamin D levels and inflammatory markers in acute stroke patients

**DOI:** 10.1002/brb3.885

**Published:** 2018-01-11

**Authors:** Qiongzhang Wang, Zhuoying Zhu, Yuntao Liu, Xinjie Tu, Jincai He

**Affiliations:** ^1^ Department of Neurology The First Affiliated Hospital of Wenzhou Medical University Wenzhou Zhejiang Province China

**Keywords:** depression symptom, inflammatory marker, interleukin‐6, stroke, vitamin D

## Abstract

**Introduction:**

Low serum vitamin D levels are associated with the development of poststroke depression (PSD). Inflammatory markers play an important role in pathophysiology of PSD. The relationship between vitamin D levels and inflammatory markers has been discussed in nonstroke individuals. The purposes of this study were to explore the relationship between vitamin D levels and inflammatory markers in acute stroke patients and examine the effect of vitamin D and inflammatory markers on PSD.

**Methods:**

A total of 152 acute stroke patients were recruited. Serum levels of 25‐hydroxyvitamin D and inflammatory markers were measured by standardized laboratory methods. Depression symptoms were assessed with the 17‐item Hamilton Depression Scale (HAMD‐17). Patients with the HAMD‐17 scores ≥7 were identified to have depression symptoms.

**Results:**

Serum vitamin D levels were negatively correlated with serum levels of interleukin‐6 and high‐sensitivity C‐reactive protein (hsCRP) (*r* = −.244, *p* = .002; *r* = −.231, *p* = .004). Multiple regression analysis showed that interleukin‐6 and hsCRP levels were associated with vitamin D levels (*B* = −0.355, *p* = .003; *B* = −2.085, *p* = .006), whereas age, height, weight, leukocyte count, neutrophil ratio, and lymphocyte rate could be omitted without changing the results. In multivariate analyses, the serum levels of vitamin D and interleukin‐6 were associated with the development of PSD after adjusted possible variables (OR = 0.976, 95% CI: 0.958–0.994, *p* = .009; OR = 1.029, 95% CI: 1.003–1.055, *p* = .027).

**Conclusions:**

Serum vitamin D levels are inversely associated with the levels of interleukin‐6 and hsCRP, suggesting a potential anti‐inflammatory role for vitamin D in stroke individuals.

## 1 INTRODUCTION

Vitamin D is a kind of micronutrient which is well known for its key role in musculoskeletal health and calcium homeostasis (Freyschuss, Ljunggren, Saaf, Mellstrom, & Avenell, 2007). Vitamin D is present in multiple forms in the body (Kulie, Groff, Redmer, Hounshell, & Schrager, 2010). Vitamin D3 is synthesized from 7‐dehydrocholesterol in the skin by way of ultraviolet (UV) B rays (Holick, 1992). The vitamin D binding protein transports the vitamin D3 to the liver where it undergoes hydroxylation to 25‐hydroxyvitamin D (25(OH)D) and then to the kidneys where it is hydroxylated by the enzyme 1 hydroxylase to 1,25(OH)D 25‐hydroxyvitamin D(1,25(OH)_2_ D) (Brannon, Yetley, Bailey, & Picciano, 2008). In addition, vitamin D is a key regulatory factor of immune function and inflammation (Gonzalez‐Molero et al., 2013; Hossein‐Nezhad et al., 2013). More and more researchers have realized the effect of vitamin D on cells of the adaptive and innate immune system (Sloka, Silva, Wang, & Yong, 2011). Accumulating evidence has suggested that the level of vitamin D may be essential for the optimal anti‐inflammatory response of monocytes (Zhang et al., 2012). The conversion of 25(OH)D to its active form 1,25(OH)_2_D occurs in immune system cells (Amento, 1987). Vitamin D has an anti‐inflammatory effect on the inflammatory profile of monocytes, downregulating the expression of several pro‐inflammatory cytokines including tumor necrosis factor‐α (TNF‐α) and IL‐6 (Giulietti et al., 2007). Vitamin D receptor (VDR) has been found in multiple cells of the immune system such as human regulatory T cells (Khoo et al., 2011), B cells (Adorini, 2003), neutrophils (Takahashi et al., 2002), dendritic cells, and macrophages (Mocanu, Oboroceanu, & Zugun‐Eloae, 2013). The process of vitamin D production is subsequently modulated by cellular activation of pro‐inflammatory cytokines (Baeten & Kuchroo, 2013). Meanwhile, vitamin D can reduce immune responses and inflammatory reaction in these processes (White, 2012). A great number of epidemiologic studies have demonstrated its effect on immune system (Prietl, Treiber, Pieber, & Amrein, 2013) and protective actions on cardiovascular system (Pilz, Verheyen, Grubler, Tomaschitz, & Marz, 2016). Low levels of vitamin D have been found to be associated with increased cardiovascular mortality (Holick, 2011), cancer incidence and mortality (Tagliabue, Raimondi, & Gandini, 2015), and autoimmune diseases such as multiple sclerosis (Alharbi, 2015), probably caused by its anti‐inflammatory and immune‐modulating properties. Acute stroke patients have been proved to have lower serum vitamin D levels compared to normal controls, and it predicts the poorer prognosis of stroke (Afshari, Amani, Soltani, Haghighizadeh, & Afsharmanesh, 2015; Alfieri et al., 2017). Besides, vitamin D is widely accepted as a necessity for normal physiological process (Daly et al., 2012). Early animal studies have implicated the contributions of vitamin D to normal brain equilibrium and physiological processes (Almeras et al., 2007; Eyles et al., 2007). Vitamin D is necessary for normal brain development and function (Eyles, Liu, Josh, & Cui, 2014). Various of psychiatric disorders such as schizophrenia (Cieslak et al., 2014), late‐life depression (Parker, Brotchie, & Graham, 2017), and anxiety (Bicikova et al., 2015) are related to low levels of vitamin D. Recently, researchers have demonstrated that low levels of vitamin D were associated with the development of poststroke depression (PSD) (Kaloglu et al., 2016) and the underlying mechanism is probably involved in its anti‐inflammatory and immune‐modulating effects (Han, Lyu, Sun, Wei, & He, 2015).

Inflammatory marker is a participant in inflammatory responses (Medzhitov, 2010), and it is involved in the development of atherosclerosis and stroke (Elkind, 2006). Leukocyte count has been proved to be independently associated with carotid plaque thickness (Elkind, Cheng, Boden‐Albala, Paik, & Sacco, 2001) and progression of carotid intima‐media thickness (Salonen & Salonen, 1990), as well as with risk of stroke (Grau, Buggle, Becher, Werle, & Hacke, 1996; Prentice, Szatrowski, Kato, & Mason, 1982). High‐sensitivity C‐reactive protein (hsCRP) has been shown to predict the occurrence of cardiovascular events in several populations (Ridker, 1999). A follow‐up study has demonstrated that elevated levels of hsCRP within 72 hr of stroke predicted increased mortality over a follow‐up period of 4 years (Arenillas et al., 2003). In animal experiments, overexpression of interleukin (IL)‐1a, IL‐1b, IL‐1 receptor antagonist (IL‐1ra), IL‐6, and TNF‐α has been found in the brain after stroke (Hill et al., 1999; Legos et al., 2000; Zhai, Futrell, & Chen, 1997). The pro‐inflammatory cytokines TNF‐α and IL‐6 are involved in the initiation or amplification of the inflammatory response (Spalletta et al., 2006), and higher levels of TNF‐α and IL‐6 have indicated the poor outcome of stroke (Tarkowski et al., 1995; Zaremba & Losy, 2001). IL‐6, but not TNF‐α, measured at baseline has been considered to be an independent predictor of worsening in the first 24 hr after stroke (Muir, Weir, Alwan, Squire, & Lees, 1999). Moreover, polymorphisms of the promoter IL‐6 gene have been proved to be associated with the presence of stroke (Pola et al., 2003; Revilla et al., 2002) while polymorphisms of the TNF gene and IL‐1 gene have been suspected to be associated with ischemic stroke (Lee et al., 2004). In addition, inflammatory marker has been considered to be associated with varies of psychiatric disorders such as bipolar disorder, schizophrenia, and depression (Raison & Miller, 2013; Scola & Andreazza, 2014; Tomasik, Rahmoune, Guest, & Bahn, 2016). Elevated levels of TNF‐α and IL‐6 in the plasma have been found in patients with major depressive disorder (Kubera et al., 2000; Tuglu, Kara, Caliyurt, Vardar, & Abay, 2003). Many studies have found significant associations between blood concentrations of inflammatory factors and the severity of depressive symptoms (Alesci et al., 2005; Bower, Ganz, Aziz, & Fahey, 2002; Miller, Stetler, Carney, Freedland, & Banks, 2002; Motivala, Sarfatti, Olmos, & Irwin, 2005), although the results of the studies are not entirely consistent. A recent study conducted by Hiles and colleagues has shown that antidepressants reduced the overproduction of inflammatory cytokines and improved depressive symptoms (Hiles, Baker, de Malmanche, & Attia, 2012). Moreover, elevated levels of IL‐6 and TNF‐α have been also found in stroke patients with depressive symptoms (Su, Chou, Tsai, & Hung, 2012). IL‐6 has been considered to be a key mediator of circulating pro‐inflammatory cytokines in pathophysiology of PSD.

Although a potential relationship between serum vitamin D levels and inflammatory markers has been hypothesized, there is inconclusive evidence about the anti‐inflammatory role of vitamin D, especially in the stroke patients. The association between serum vitamin D levels and multiple inflammatory markers has been addressed in few human studies. Only one study focused on the relationship between the serum vitamin D levels and inflammatory markers in acute ischemia stroke individuals has not taken into account the complete role of different inflammatory markers such as neutrophil‐to‐lymphocyte ratio (Alfieri et al., 2017). Besides, the study conducted in acute ischemia stroke patients ignored the depression symptoms after stroke. Given the anti‐inflammatory profile of vitamin D and its effect on development of PSD, we assessed whether serum vitamin D levels were inversely associated with the blood concentration of inflammatory markers in acute stroke patients and examined the effect of vitamin D and inflammatory markers on PSD.

## 2 METHODS

### 2.1 Study population

Patients with acute stroke were admitted to the stroke unit between January 2014 and January 2015. This study protocol was approved by the Medical Ethics Committee of the First Affiliated Hospital of Wenzhou Medical University. The informed consents were signed by the patients and their relatives. The research was performed according to the principles expressed in the Declaration of Helsinki. The patients were prohibited to take antidepressants during the acute period of stroke. The inclusive criteria were listed as follows: (1) age 18–80 years; (2) acute stroke occurring within 72 hr before admission; (3) diagnosed by computerized tomography (CT) or magnetic resonance imaging (MRI); (4) the ability and willingness to provide information; (5) with complete information including serum levels of vitamin D, hsCRP, leukocyte count, neutrophil‐to‐lymphocyte ratio, IL‐6 and TNF‐α. We excluded the patients who met the following criteria: (1) with infectious diseases or hsCRP ≥10 mg/dl; (2) those who were on chronic treatments with corticosteroids and nonsteroidal inflammatory drugs; (3) with a history of major depression (clinical diagnosis or previous treatment) or other psychiatric disorders; (4) with severe aphasia; (5) with a history of severe nervous system diseases such as dementia, Parkinson's disease, trauma or hydrocephalus; (6) with multiple organ failure or tumor; (7) in severe condition that they could not finish the scales.

### 2.2 Laboratory test

Blood samples were obtained in the first morning at admission after a 12‐hr overnight fast and resting period. The blood samples were divided into two parts. One of the two parts was stored at −80°C while the others were measured immediately. IL‐6 and TNF‐α assays were performed on specimens previously stored at −80°C, while other biological markers assays were performed on fresh blood. IL‐6 and TNF‐α assays were carried out by professional physician blinded to the scale scores at Shanghai Biochemical Laboratory, while other biological markers assays were carried out by professional physician blinded to the scale scores at our hospital's biochemical laboratory. We chose serum 25(OH)D as the index of vitamin D status for all of the subjects. Serum levels of 25(OH)D were tested by a competitive protein‐binding assay. The intra‐assay coefficient of variation (CV) was 7%–10%. Leukocyte count and neutrophil‐to‐lymphocyte ratio were measured by automatic blood cell counter at our hospital's biochemical laboratory, and hsCRP levels were measured by immunoturbidometric assay method. The interassay CV was 5%. Serum levels of IL‐6 and TNF‐α were measured in duplicate high sensitivity enzyme‐linked immunoabsorbent assays (ELISA). The interassay CV for the IL‐6 was 7%. The intra‐ assay and interassay CVs for TNF‐α were 7% and <20%, respectively.

### 2.3 Scale measurement

The severity of stroke was assessed by the National Institutes of Health Stroke Scale (NIHSS) at admission. Cognitive functions were evaluated using the Mini‐Mental State Examination (MMSE) at 3–5 days after admission to hospital. Depression symptoms were screened by the 17‐item Hamilton depression Rating Scale (HAMD) at 1 month. The patients with a score of HAMD‐17 ≥7 were diagnosed as PSD. All of the scales were performed by the researchers, who were blinded to laboratory results.

### 2.4 Other measures

Demographic data (age, gender, education years, height, weight, body mass index, etc.), lifestyle characteristics (smoking status, alcohol intake, etc.), and health status (hypertension, diabetes mellitus, hyperlipidemia, coronary disease, history of stroke, etc.) were collected by trained physicians in 3–5 days after admission. Brain CT/MRI scans were performed by professional radiologists at admission.

### 2.5 Statistical analyses

Results were exhibited as mean ± standard deviation (*SD*) and median (median‐ interquartile range, median + interquartile range) for continuous variables depending on the normal or non‐normal distribution of data, while categorical variables were expressed as number (percentage). Student's *t* test was used to explain the differences between the normally distributed variables, while the Mann–Whitney *U* test was used to the non‐normal distributed variables. The categorical variables were compared using the chi‐squared test. Factors statistically correlated with vitamin D levels were identified by Pearson rank correlation coefficient and Spearman rank correlation coefficients as appropriate. Multiple linear regression analysis including all factors significantly different in the correlation analysis was performed to find the possible potential determining factors of vitamin D levels. Binary logistic regression including all factors significantly different in the univariate analysis was performed to determine significant risk factors of the development of PSD. The results were expressed as adjusted odds ratios (ORs) with the corresponding 95% confidence intervals (CIs). All statistical tests were performed with SPSS for Windows (Release 19.0; SPSS, Chicago, IL, USA). Values of *p* < .05 were considered to be statistically significant in all tests.

## 3 RESULTS

In this study, a total of 187 individuals met the entry criteria and were admitted to the stroke unit. Thirty‐five patients were lost to follow‐up. Complete data were obtained from 152 patients. The lost rate of follow‐up was 18.7%. There was no difference in NIHSS scores between the patients included in the study and the patients lost to follow‐up (2 [1–4] vs. 3 [1–4], *Z* = −0.100, *p* = .920).

We found that vitamin D levels were inversely correlated with serum IL‐6 levels and hsCRP (*r* = −.244, *p* = .002; *r* = −.231, *p* = .004) while serum vitamin D levels were not significantly correlated with other inflammatory markers such as leukocyte count, neutrophil‐to‐lymphocyte ratio, and TNF‐α. In addition, we did not find significant correlation between age and vitamin D levels while female sex, history of diabetes mellitus, and drinking were correlated strongly with serum vitamin D levels (*r* = −.175, *p* = .031; *r* = −.263, *p* = .001; *r* = .166, *p* = .040) (Table 1). In multivariate linear regression, vitamin D levels were negatively associated with the levels of IL‐6 and hsCRP after adjustment for the above variables. (*B* = −0.335, *p* = 0.003; *B* = −2.085, *p* = .006) (Table 2).

**Table 1:** Correlation coefficients evaluating the relationship of 25‐hydroxyvitamin D and inflammatory markers and other potential confounding variables

Vitamin D (nmol/L)	*r*	*p* Value
Age (years)	.070	.390
Female sex	−.175	.031
Height (cm)	.070	.390
Weight (kg)	−.056	.494
BMI (kg/m^2^)	−.132	.104
Education years	−.150	.065
SBP (mmHg)	−.006	.943
DBP (mmHg)	.054	.506
Vascular risk factors		
Hypertension	−.060	.466
Diabetes mellitus	−.263	.001
CAD	.030	.712
Hyperlipidemia	.028	.730
History of stroke	−.116	.155
Current smoking	.041	.616
Current drinking	.166	.040
Laboratory findings		
hsCRP (mg/L)	−.231	.004
Leukocyte count	.044	.589
Neutrophil ratio	.055	.498
Lymphocyte rate	−.124	.128
Neutrophil‐to‐lymphocyte ratio	.097	.235
IL‐6 (pg/ml)	−.244	.002
TNF‐α (pg/ml)	.048	.559

BMI, body mass index; CAD, coronary artery disease; DBP, diastolic blood pressure; hsCRP, high‐sensitivity C‐reactive protein; IL‐6, interleukin‐6; SBP, systolic blood pressure; TNF‐α, tumor necrosis factor‐α.

**Table 2:** Linear regression analysis of the relationship between inflammatory marker and vitamin D levels

	Unstandardized coefficients		95.0% CI for *B*		*p* Value
	*B*	Std error	Lower bound	Upper bound	
Female sex	−8.579	3.620	−15.733	−1.425	.019
Diabetes mellitus	−12.592	4.587	−21.657	−3.527	.007
Current drinking					.535
hsCRP	−2.085	0.752	−3.571	−0.600	.006
IL‐6	−0.335	0.110	−0.552	−0.118	.003

CI, confidence intervals; hsCRP, high‐sensitivity C‐reactive protein; IL‐6, interleukin‐6; Std, standardized.

Characteristics of the 152 stroke patients were displayed in Table 3. In this study, participants were 63 (41.4%) females, and the median (median − interquartile range [IQR], median + IQR) age was 65.00 (56.00–70.75) years old. The median (median − IQR, median + IQR) score of NIHSS on our study was 2 (1–4). There were 45 (29.6%, 21 male, 24 female) patients diagnosed as PSD. The serum levels of vitamin D were significantly lower in PSD group than non‐PSD group (44.27 ± 24.04 nmol/L vs. 57.64 ± 23.22 nmol/L, *t* = 3.208, *p* = .002). Moreover, serum levels of IL‐6 and TNF‐α were significantly higher in PSD group than non‐PSD group (58.25 ± 16.39 pg/ml vs. 48.77 ± 15.57 pg/ml, *t* = −3.372, *p* = .001; 315.45 ± 97.50 pg/ml vs. 268.41 ± 103.97 pg/ml, *t* = −2.593, *p* = .01), while hsCRP and neutrophil‐to‐lymphocyte ratio were not significantly different between the two groups. In addition, PSD group had higher scores of NIHSS and more leukocyte count (3 [2–4.5] vs. 2 [1–3], *Z* = −2.383, *p* = .017; 6.94 ± 1.62 vs. 6.34 ± 1.64, *t* = −2.086, *p* = .039) (Table 3). In the logistic regression analysis, the serum levels of vitamin D and IL‐6 were associated with the development of PSD at 1 month (OR = 0.976, 95% CI: 0.958–0.994, *p* = .009; OR = 1.029, 95% CI: 1.003–1.055, *p* = .027) after adjustment for NIHSS scores, serum TNF‐α levels, and leukocyte count. Moreover, the NIHSS scores at admission were significantly associated with the presence of depression symptoms after stroke (OR = 1.178; 95% CI: 1.001–1.386; *p* = .048) (Table 4).

**Table 3:** Baseline clinical characteristics in PSD and non‐PSD

	Non‐PSD (*n* = 107)	PSD (*n* = 45)	*p* Value
Age (years), median (median − IQR, median + IQR)	65 (58–71)	65 (52–70.5)	.413
Female sex, *n* (%)	39 (36.4)	24 (53.3)	.054
Height (cm), mean ± *SD*	163.79 ± 7.21	161.84 ± 8.31	.150
Weight (kg), mean ± *SD*	64.77 ± 9.35	64.77 ± 11.31	.998
BMI (kg/m^2^), mean ± *SD*	24.09 ± 2.63	24.65 ± 3.43	.272
Education years, median (median − IQR, median + IQR)	3 (0–6)	5 (0–8)	.185
SBP (mmHg), mean ± *SD*	156.10 ± 20.78	152.93 ± 24.41	.417
DBP (mmHg), mean ± *SD*	83.10 ± 12.82	80.33 ± 13.14	.229
Vascular risk factors, *n* (%)			
Hypertension	85 (79.4)	29 (64.4)	.051
Diabetes mellitus	21 (19.6)	9 (20.0)	.958
CAD	8 (7.5)	1 (2.2)	.381
Hyperlipidemia	4 (3.7)	3 (6.7)	.717
History of stroke	9 (8.4)	8 (17.8)	.094
Current smoking	30 (28.0)	11 (24.4)	.649
Current drinking	35 (32.7)	13 (28.9)	.644
Neuropsychological function			
NIHSS score at baseline, median (median − IQR, median + IQR)	2 (1–3)	3 (2–4.5)	.017
MMSE score at baseline, median (median − IQR, median + IQR)	24 (19–27)	23 (16.5–25.5)	.437
Laboratory findings			
hsCRP (mg/L), median (median − IQR, median + IQR)	1.07 (0.54–3.07)	2.62 (0.68–4.74)	.066
Leukocyte count, mean ± *SD*	6.34 ± 1.64	6.94 ± 1.62	.039
Neutrophil ratio, median (median − IQR, median + IQR)	0.58 (0.53–0.65)	0.60 (0.52–0.68)	.338
Lymphocyte rate, median (median − IQR, median + IQR)	0.30 (0.25–0.36)	0.29 (0.23–0.36)	.372
Neutrophil‐to‐lymphocyte ratio	2.04 (1.45–2.56)	1.97 (1.58–2.80)	.442
IL‐6 (pg/ml), mean ± *SD*	48.77 ± 15.57	58.25 ± 16.39	.001
TNF‐α (pg/ml), mean ± *SD*	268.41 ± 103.97	315.45 ± 97.50	.010
Vitamin D (nmol/L), mean ± *SD*	57.64 ± 23.22	44.27 ± 24.04	.002

BMI, body mass index; CAD, coronary artery disease; DBP, diastolic blood pressure; hsCRP, high‐sensitivity C‐reactive protein; IL‐6, interleukin‐6; IQR, interquartile range; MMSE, Mini‐Mental State Examination; NIHSS, National Institutes of Health Stroke Scale; PSD, poststroke depression; SBP, systolic blood pressure; *SD*, standard deviation; TNF‐α, tumor necrosis factor‐α.

Values are shown as number (percentage) or as median (median − IQR, median + IQR) and mean ± *SD*.

**Table 4:** Multivariate logistic model of the clinical determinants of PSD

Variables	OR	95% CI	*p* Value
NIHSS score at baseline	1.178	1.001–1.386	.048
Leukocyte count	1.287	1.001–1.656	.050
IL‐6	1.029	1.003–1.055	.027
TNF‐α			.079
Vitamin D	0.976	0.958–0.994	.009

CI, confidence intervals; IL‐6, interleukin‐6; NIHSS, National Institutes of Health Stroke Scale; OR, odds ratios; PSD, poststroke depression; TNF‐α, tumor necrosis factor‐α.

## 4 DISCUSSION

To our knowledge, this is the first study to assess the relationship between serum vitamin D levels and inflammatory makers including hsCRP, leukocyte count, neutrophil‐to‐lymphocyte ratio, IL‐6 and TNF‐α in acute stroke patients. We found that serum vitamin D levels were inversely associated with the serum IL‐6 levels in acute stroke patients. Moreover, we found that depression symptoms after stroke were associated with the low serum vitamin D levels and elevated serum IL‐6 levels, which indeed provides evidence for the hypotheses about the underlying mechanism of PSD.

No significant correlation between vitamin D levels and age was found, which is not consistent with some previous studies (Serdar et al., 2017). The previous studies insisted on that the serum levels of vitamin D were decreasing with the growth of age (Bani‐issa, Eldeirawi, Harfil, & Fakhry, 2017). Serum levels of vitamin D were tested in different age groups including the young and old population among these studies, while the subjects of our study were aged. This might be an important explanation for our different results. A random population study conducted in the individuals aged 35–65 years showed that there was no correlation between vitamin D levels and age (Rudnicki, Thode, Jorgensen, Heitmann, & Sorensen, 1993). Interestingly, we found that female individuals seemed to have lower serum levels of vitamin D in our studies, while some studies showed that there was no significant correlation between sex and vitamin D deficiency (Bani‐issa et al., 2017). Most of these studies were carried on Western country in which taking part in outdoor exercise is encouraged. In china, female is not willing to taking part in outdoor exercise and always takes action to protect from UV, which might contribute to the lower serum vitamin D levels in female. We also found that patients with a history of diabetes had lower serum levels of vitamin D, which is consistent with previous studies (Berridge, 2017).

In fact, the relationship between serum vitamin D levels and inflammatory markers has been discussed in nonstroke patients. Few evidence coming from observational studies supports a primary anti‐inflammatory role of vitamin D (Azizieh, Alyahya, & Dingle, 2017; De Vita et al., 2014; Donate‐Correa et al., 2017; Mateen, Moin, Shahzad, & Khan, 2017). An observational investigation conducted in 957 old individuals, Laird et al. (2014) demonstrated a significant association between low vitamin D status (25OH‐D < 25 nmol/L) and markers of inflammation including IL‐6, hsCRP and the ratio of IL‐6 to IL‐10. Ngo, Sverdlov, McNeil, and Horowitz (2010), in a cohort of 253 middle‐aged and older subjects, have found that low vitamin D levels were inversely associated with hsCRP levels, which is consistent with our results. Another observational study conducted in rheumatoid arthritis patients has found that serum vitamin D levels were negatively correlated with the levels of IL‐6 and TNF‐a (Mateen et al., 2017). Similar to previous results, an inverse relationship between serum vitamin D levels and IL‐13 levels was documented by Bilir et al. (2016) in population of diabetic peripheral neuropathy. Previous study has indicated that the immune–inflammatory activation could be involved in the pathogenesis of the arterial stiffness and acute ischemia (Tuttolomondo et al., 2012). Moreover, higher peripheral frequency of CD4(+) CD28(null) T cells has been found in acute ischemic stroke patients compared to nonstroke patients (Tuttolomondo et al., 2015). However, the study focused on the relationship between vitamin D and inflammatory markers in acute stroke patients is rare. A recent study which has been designed to explore the relationship between serum vitamin D and inflammatory markers in acute ischemic stroke patients showed that vitamin D deficiency (25OH‐D < 20 nmol/L) was significantly associated with hsCRP and short‐term outcome (Alfieri et al., 2017).

On the contrary, several studies failed to prove any significant association between serum vitamin D levels and the inflammatory markers (Clendenen et al., 2011; Gannage‐Yared et al., 2003; Shea et al., 2008; Vilarrasa et al., 2010). Interestingly, scarce attention has been devoted to the relationship between vitamin D and IL‐6. Dobnig et al. (2008), in a prospective cohort study of more than 3,000 consecutive patients undergoing coronary angiography, has announced that low vitamin D levels were significantly correlated with the levels of IL‐6 and CRP. Consistently, an inverse relationship between serum vitamin D levels and IL‐6 levels was documented by De Vita et al. (2014) in 867 older adults. Paricalcitol, a selective VDR activator, has been found to reduce serum levels of IL‐6 and TNF‐α (Donate‐Correa et al., 2017).

The molecular mechanisms underlying the relationship between vitamin D and IL‐6 could be considered to be involved in the inhibitory action of vitamin D on cellular expression (Giulietti et al., 2007; Neve, Corrado, & Cantatore, 2014). The pro‐inflammatory transcription factor kappa B (NFκB) and MAPK phosphatase‐1 (MKP‐1) has been considered to play an important role in this process (Neve et al., 2014; Zhang et al., 2012).Vitamin D has been proved to regulate the release of the inflammatory cytokine IL‐6, which represents a downstream target of NFκB activation (Jablonski, Chonchol, Pierce, Walker, & Seals, 2011; Suzuki et al., 2009). VDR, which is a part of the inactivating complex with the nuclear p65 subunit of NFκB (Equils et al., 2006), may play a role in this process. Studies conducted in mice have shown that VDR deletion abolished VDR/P65 binding can contribute to upregulation of NFκB transcriptional activity and increase IL‐6 circulating levels (Wu et al., 2010). The MAPKs activated by LPS are critical regulators of pro‐inflammatory cytokine production, including TNF‐α and IL‐6 (Bhavsar et al., 2008; Kracht & Saklatvala, 2002). LPS, a component of the Gram‐negative bacterial cell wall, induces cytokine production by monocytes and macrophages (Zhang et al., 2012, 2012). Vitamin D inhibition of LPS‐induced IL‐6 and TNF‐α production by bone marrow‐derived macrophages from MKP‐1 deletion mice was significantly reduced as compared with wild‐type mice (Zhang et al., 2012).

A negative relationship between vitamin D and hsCRP was found in our study, which is consistent with previous studies (Alfieri et al., 2017). A cross‐sectional study of 64 obese children aged 6–16 years has shown that obese children with vitamin D deficiency had higher levels of hsCRP (Ngo et al., 2010). Meanwhile, the inverse relationship between serum vitamin D status and hsCRP levels was documented by Haidari, Jalali, Shahbazian, Haghighizadeh, and Azadegan (2016) in population of gestational diabetes mellitus and normal glucose tolerance pregnant women. In addition, no significant correlation between hsCRP and PSD was found in the study, which is similar to the previous studies (Jimenez et al., 2009).

We failed to detect any association between vitamin D and TNF‐α, which is consistent with the previous study conducted in acute ischemia stroke (Alfieri et al., 2017). However, an observational study included 69 healthy women showed that vitamin D levels were independently inversely associated with TNF‐α levels (Peterson & Heffernan, 2008). Moreover, an investigation conducted in monocytes from type 2 diabetic patients showed that 1,25‐dihydroxyvitamin D (3) was able to downregulate the expression of TNF‐α, IL‐6, IL‐1, and IL‐8 (Giulietti et al., 2007). Vitamin D deficiency has been proved to be involved in enhanced expression of TNF‐α in mice (Li et al., 2014), while another observational investigation conducted in 957 old individuals showed that there was no association between vitamin D and TNF‐α levels. More studies are needed to fully evaluate the relationship between TNF‐α system and vitamin D in stroke patients. Besides, no significant correlation between vitamin D and leukocyte count was found in our study, which is consistent with the previous studies in nonstroke patients (Lee, Kim, Lim, & Hong, 2015; Yildirim, Hur, & Kokturk, 2013). In addition, our results showed that the neutrophil‐to‐lymphocyte ratio was also not significant correlated with vitamin D in stroke patients, while a recent investigation conducted in adolescent girls has observed a significant reduction in neutrophil‐to‐lymphocyte ratio after high‐dose vitamin D supplementation (Tabatabaeizadeh et al., 2017). Another observational study conducted in children with community‐acquired pneumonia (CAP) has found that vitamin D deficiency had a significantly higher neutrophil‐to‐lymphocyte ratio distribution (Huang, Fu, & Yang, 2017). The relationship between neutrophil‐to‐lymphocyte ratio and vitamin D has not been discussed in stroke patients before the study. To explain this specific issue, we tested the relationship between vitamin D levels and neutrophil ratio. Meanwhile, the correlation between vitamin D levels and lymphocyte rate was tested in our study. However, we did not detect any significant correlation between them, suggesting that vitamin D might not affect the neutrophil ratio and lymphocyte rate in stroke patients. Of course, more investigations are needed to fully evaluate the relationship between vitamin D and neutrophil‐to‐lymphocyte ratio in stroke patients.

Finally, we examined the effect of vitamin D and IL‐6 on PSD. We found that vitamin D and IL‐6 were associated with the development of PSD. Similar to our result, low level of vitamin D has been considered as an important predicting factor for PSD (Han et al., 2015; Kaloglu et al., 2016) and IL‐6 has been showed to play a vital role in pathophysiology of PSD (Spalletta et al., 2013). Researchers have advanced one hypothesis that anti‐inflammatory role of vitamin D may play a role in the pathophysiology of PSD (Han et al., 2015). A recent study has demonstrated that the use of stain and liver‐soothing‐oriented method prevented from PSD by decreasing the levels of IL‐6 (Kang et al., 2016; Zeng et al., 2016). However, the studies aimed to reveal the mechanisms of PSD in clinical populations are still rare (Robinson & Jorge, 2016). Our finding is of importance because it provides evidence for the underlying mechanism of PSD. Unfortunately, the nature of our study does not allow us to determine the direction of the association between vitamin D and inflammatory markers. In fact, low vitamin D status and higher levels of inflammatory marker are widely reported in a wide range of disorders such as multiple sclerosis (Bhargava et al., 2014; Patejdl & Zettl, 2017), schizophrenia (Cieslak et al., 2014) (Aas et al., 2017), and anxiety (Bicikova et al., 2015; Memon et al., 2017). A large amount of evidence is needed to prove that the elevated levels of vitamin D can modify the occurrence or clinical course of diseases is not causal and reveal the potential mechanism of these disorders.

Some limitations of the study must be recognized. First, because of the nature of the study, we cannot define cause–effect relationships between vitamin D and inflammatory markers. Second, the information for dietary intake and sunlight hours was not recorded which may produce the bias. Third, we excluded the patients in severe condition and patients with severe aphasia, which may produce selection bias. Fourth, the number of research subjects is a little bit small although this is a very compelling study.

In conclusion, our results demonstrated that the associations between serum vitamin D and IL‐6 levels in stroke patients, suggesting a potential anti‐inflammatory role for vitamin D. It indeed provides evidences for the pathophysiology of PSD caused by vitamin D deficiency. If it is confirmed by longitudinal studies, the results may better delineate the direction of the association between vitamin D and IL‐6. Randomized controlled studies, examining the effects of vitamin D administration on inflammatory markers and clinical outcomes are clearly needed.

## CONFLICT OF INTEREST

None declared.
